# Novel Classification of the Posterior Auricular Artery Based on Angiographical Appearance

**DOI:** 10.1371/journal.pone.0128723

**Published:** 2015-06-01

**Authors:** Joji Tokugawa, Narisumi Cho, Hiroharu Suzuki, Natsuki Sugiyama, Osamu Akiyama, Yasuaki Nakao, Takuji Yamamoto

**Affiliations:** 1 Department of Neurosurgery, Juntendo University Shizuoka Hospital, Izunokuni, Shizuoka, Japan; 2 Department of Radiology, Juntendo University Shizuoka Hospital, Izunokuni, Shizuoka, Japan; University of Washington School of Medicine, UNITED STATES

## Abstract

**Purpose:**

To investigate the length variation of the posterior auricular artery and propose a novel classification of the posterior auricular artery based on angiographical appearance.

**Patients and Methods:**

A series of 234 consecutive patients who had undergone conventional cerebral angiography was analyzed. The posterior auricular artery was examined on the lateral projection of the external carotid or common carotid arteriography. The posterior auricular artery was classified into four groups by length, using the external auditory canal and the top of the helix as radiographical landmarks. Our proposed classification is as follows: Type A, posterior auricular artery terminates between its origin and the center of the external auditory canal; Type B, posterior auricular artery terminates between the center of the external auditory canal and the top of the helix; Type C, posterior auricular artery terminates between the top of the helix and the vertex; and Type D, posterior auricular artery reaches up to the vertex.

**Results:**

A total of 424 (right, 214; left, 210) posterior auricular arteries were analyzed in 111 men and 123 women aged 11 to 91 years (mean, 61.0 years) examined for aneurysms in 78 cases, occlusive vascular diseases in 56, intracranial hemorrhages in 41, tumors in 35, and others in 24. Types A, B, C, and D were found in 15.1%, 34.9%, 48.8%, and 1.2% of the patients, respectively.

**Conclusion:**

A novel classification of the posterior auricular artery identifies four types based on its length on cerebral angiography.

## Introduction

The posterior auricular artery (PAA) is recognized as one of the small branches arising from the external carotid artery and supplies a relatively small area of the skin posterior to the ear and the ear itself [1,2]. Anatomical consideration of this artery is quite limited in the standard neuroradiographical text books [3,4]. Conventional cerebral angiography generally shows a slender PAA, but we recently treated a patient with an exceptionally large PAA which was suitable as a donor artery for cerebral revascularization surgery [5]. From this case, we speculated that the PAA may have considerable variation in length.

The present study investigated the variation in angiographical length of the PAA and proposes a novel classification of the PAA based on angiographical appearance.

## Patients and Methods

**Fig 1:**
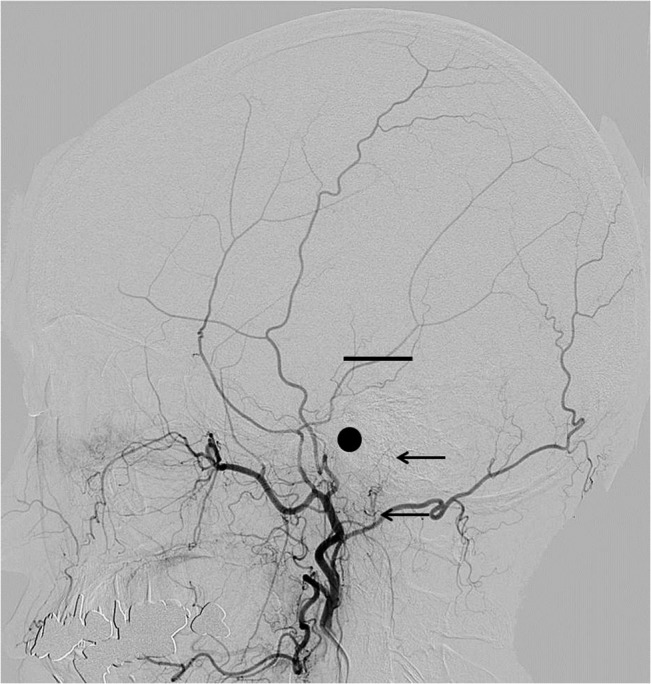
Representative cases of PAA: Type A. Representative cases of PAA in each type are shown with black arrows. In each figure, a black dot shows the external auditory canal and a short straight line shows the top of the helix.

**Fig 2:**
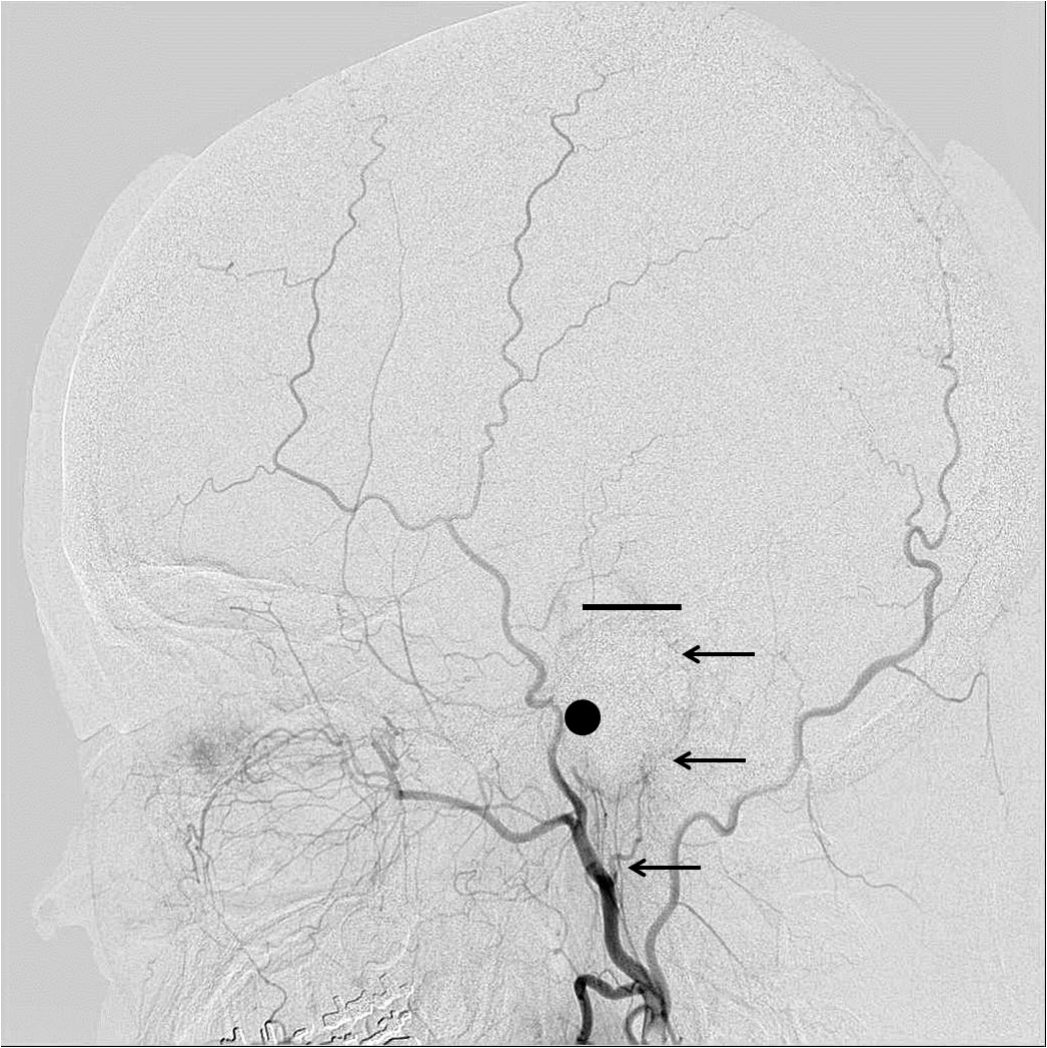
Representative cases of PAA: Type B. Representative cases of PAA in each type are shown with black arrows. In each figure, a black dot shows the external auditory canal and a short straight line shows the top of the helix.

**Fig 3:**
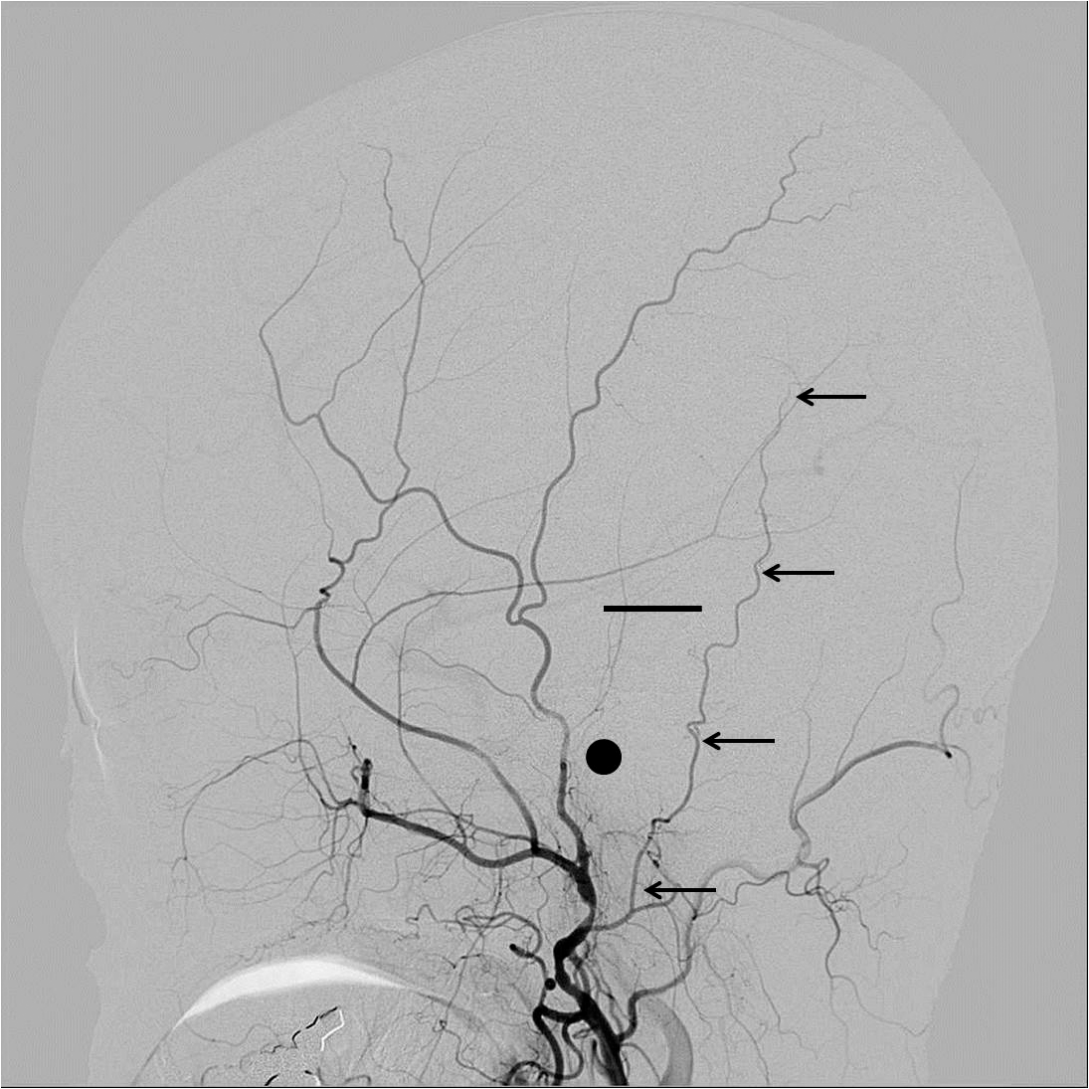
Representative cases of PAA: Type C. Representative cases of PAA in each type are shown with black arrows. In each figure, a black dot shows the external auditory canal and a short straight line shows the top of the helix.

**Fig 4:**
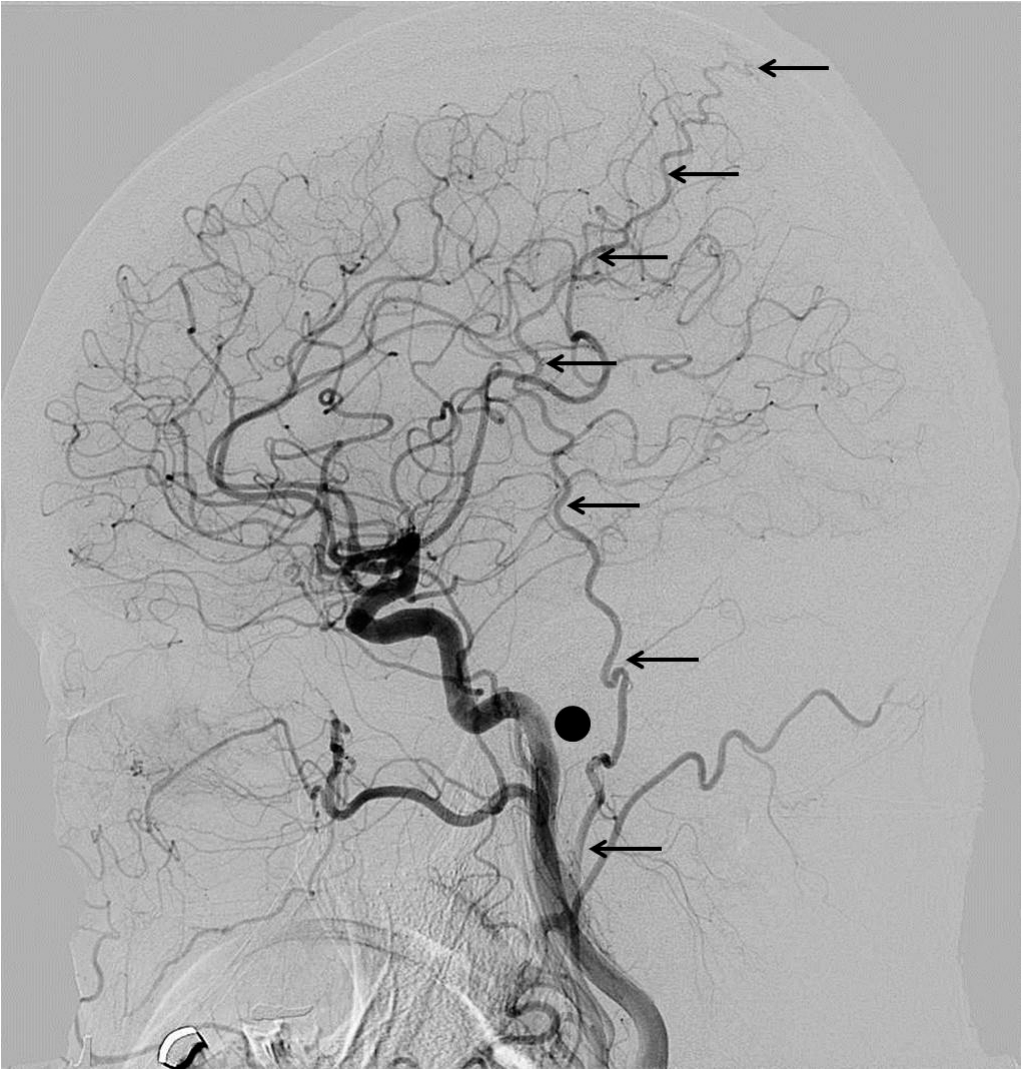
Representative cases of PAA: Type D. Representative cases of PAA in each type are shown with black arrows. In each figure, a black dot shows the external auditory canal and a short straight line shows the top of the helix.

The present study included 234 consecutive patients who had undergone conventional cerebral angiography in our institute. The PAA was observed on the lateral projection of the conventional external carotid or common carotid arteriography. Cases were excluded of PAAs ipsilateral to the history of major head injury or laceration of the head skin, patients with tumors with possible blood supply from the external carotid artery such as meningiomas, and patients with history of craniotomy, because these conditions may cause change in the balance of the distribution of the blood supply to the skin. The PAAs were classified into four groups by length, using the external auditory canal and the top of the helix as the radiographical landmarks. This classification did not consider the size or the diameter of the PAA. The PAA was observed on cine angiography until disappearance at the end of the arterial phase to ensure evaluation of the full length of the artery. Our proposed classification is as follows: Type A, PAA terminates between the origin and the center of the external auditory canal; Type B, PAA terminates between the origin and the top of the helix; Type C, PAA terminates between the top of the helix and the vertex; and Type D, PAA reaches up to the vertex. Representative cases of each type are shown in Figs 1–4.

### Ethics statement

All data was collected retrospectively, so no patients were exposed to ionizing radiation purely for the purpose of this study. Patient records and information were anonymized and de-identified prior to analysis and the study was approved by the institutional ethics committee of Juntendo University Shizuoka Hospital. The individual patients used as representative cases in this manuscript have given written informed consent as outlined in the PLOS consent form to publish their angiographical images.

## Results

A total of 424 (right, 214; left, 210) PAAs were analyzed in 111 men and 123 women aged 11 to 91 years (mean, 61.0 years) examined for unruptured aneurysms in 78 cases, occlusive vascular diseases in 56, intracranial hemorrhages in 41, tumors in 35, arteriovenous malformations or fistulas in 14, traumas 8, and miscellaneous diseases in 2. The total number of PAAs in each type were as follows: 64 (15.1%) in Type A, 148 (34.9%) in Type B, 207 (48.8%) in Type C, and 5 (1.2%) in Type D (Table 1). Type A was short and slender, and sometimes so faint that cine angiography was needed for identification. Type B was more obvious than Type A but still slender. Type C was easy to identify but not as large as the superficial temporal artery (STA) or occipital artery (OA). Type D was as large as, or sometimes larger, than the STA or OA. The PAA varied in length, but only 5 of the 424 PAAs (1.2%) were as large as the STA. The age distribution of patients in each type is shown in Table 2. There was no significant difference in mean age between patients with the different types (one-way analysis of variance).

**Table 1:** Numbers of PAA in each type.

Type	Number (%)
**A**	64 (15.1)
**B**	148 (34.9)
**C**	207 (48.8)
**D**	5 (1.2)
**Total**	424 (100)

**Table 2:** Age distribution in each type.

Type	Age Range (y)	Mean Age (y)
**A (n = 64)**	12–78	61.6
**B (n = 148)**	13–91	59.2
**C (n = 207)**	11–91	58.2
**D (n = 5)**	44–64	52.4

## Discussion

The PAA has been examined in a number of anatomical studies [1,2,6–9]. Two studies were more focused and exact on the territory of PAA. Ink injection, radiographic examination, and latex injection were performed on a total of eight cadavers [1]. Bilateral injection of colored latex was performed on 24 cadavers [2]. These cadaver studies were well-organized and achieved reliable results, but examined only a limited number of the PAAs. We examined 424 PAAs, a number large enough to likely include all types of variation. According to our findings, Types B and C accounted for 83.7% of all cases, so represent the “normal” or “majority” angiographical presentation. This finding agrees with the results of the ink injection in cadaver study [1]. Type D was probably not identified because of the lower incidence of this length variation.

The present findings may be important in the fields of plastic surgery and neurosurgery. In plastic surgery, the PAA is frequently used for a pedicled skin flap [10–12]. If the patient has PAA of Type A, a large skin flap of this area may not be a good solution. On the other hand, if the patient has Type C or D, a larger skin flap should result in a good result. In the neurosurgical field, if the PAA is large enough such as Type D, the artery could be used as a donor artery for revascularization surgery. We previously treated a patient with a rare case of PAA-middle cerebral artery (MCA) anastomosis instead of STA-MCA anastomosis because the STA parietal branch was absent in this patient [5].

The present study found that Types A and B are too small for use as a donor artery, and no case of Type C was larger than the STA. However, four of the five Type D cases had equivalent size to the STA, and one was obviously larger than STA. Following our case report, three more similar case reports were published [13]. A series of consecutive 175 patients who received bypass surgery found that 5.7% of patients had PAAs large enough for use as a donor artery for revascularization surgery, which corresponds to Type D [13]. This five-fold difference from the present finding of 1.2% might be the result of selection bias, as all patients underwent bypass surgery. Furthermore, bypass surgery candidates may develop a larger PAA in response to cerebral blood flow limitations. Even our finding of 1.2% might be higher than the incidence in the overall population, as any patients who have to undergo any type of cerebrovascular investigation, such as conventional cerebral angiography, computed tomography angiography, or magnetic resonance angiography, may have developed a larger PAA. However, the finding that a large PAA of Type D could be a suitable donor remains valid. Moreover, such a large PAA could act as a donor artery for the MCA territory or for the posterior circulation territory.

Definitive discussion of the function of the extralarge PAA requires more data to establish the relationships between the major arteries including the STA, PAA, and OA in the distribution of the skin.

## Conclusion

A novel classification of the PAA into four types based on angiographical length found more than 80% of the arteries were Type B or C, whereas the extralarge Type D was as rare as 1%. Type D PAA is of considerable interest in certain clinical situations.
